# Pemphigus Oral Lesions Intensity Score (POLIS): A Novel Scoring System for Assessment of Severity of Oral Lesions in Pemphigus Vulgaris

**DOI:** 10.3389/fmed.2020.00449

**Published:** 2020-09-02

**Authors:** Tekumalla Sindhuja, Dipankar De, Sanjeev Handa, Sonu Goel, Rahul Mahajan, Kamal Kishore

**Affiliations:** ^1^Department of Dermatology and Venereology, All India Institute of Medical Sciences, New Delhi, India; ^2^Department of Dermatology, Venereology and Leprology, Postgraduate Institute of Medical Education and Research, Chandigarh, India; ^3^School of Public Health, Postgraduate Institute of Medical Education and Research, Chandigarh, India; ^4^Department of Biostatistics, Postgraduate Institute of Medical Education and Research, Chandigarh, India

**Keywords:** pemphigus vulgaris, oral lesions, scale development, validity, reliability, new scale

## Abstract

**Background:** Many patients with pemphigus vulgaris (PV) in India present with predominant/exclusive oral mucosal lesions. Current validated scoring systems for pemphigus do not adequately represent the clinical variability of oral lesions.

**Objective:** To develop and validate a novel scoring system exclusively for oral lesions in PV.

**Methods:** In this cross-sectional study, the Delphi method was used to build an initial scale that was administered in 115 patients with PV. Exploratory factor analysis was used to examine the underlying factor structure of the new scale. The psychometric properties of the new scale were studied. Correlations between the new scale and Autoimmune Bullous Skin Disorder Intensity Score (ABSIS), Pemphigus Disease Area Index (PDAI), and Physician Global Assessment (PGA) were also assessed.

**Results:** Content validity of the initial scale was established with an average content validity index (CVI) of 0.8. Exploratory factor analysis resulted in a 3-factor structure with a total of 9 items. Corrected item-total correlation, a measure of data quality, was more than 0.30 for all items in the new oral mucosal scale—Pemphigus Oral Lesions Intensity Score (POLIS). Significant correlations were observed between POLIS and oral ABSIS (*r* = 0.85, *p* < 0.001), mucosal PDAI (*r* = 0.70, *p* < 0.001), and PGA (*r* = 0.60, *p* < 0.001). POLIS was also reliable with good internal consistency (Cronbach's α = 0.86) and strong inter-rater agreement.

**Limitations:** The study cohort included participants from a single center. Usability and time taken to administer the scale were not assessed.

**Conclusions:** The new scale, POLIS, has adequate validity and reliability. It includes both quality of life and clinical disease severity parameters, assessing disease severity holistically. Further studies evaluating the scale's responsiveness to change are in progress.

## Introduction

Pemphigus is common in the Indian population, and they display distinct clinical and epidemiological qualities compared to western society (1). Oral mucosa is often the first and the only site to be involved in a significant proportion of patients with pemphigus vulgaris (PV) (1). Deep crater-like lesions, erosions with lichenoid hue, and lesions located on the retromolar trigone and occlusion line of buccal mucosa are refractory to treatment (2). Oral lesions tend to persist longer, require prolonged treatment course, and are associated with increased morbidity, leading to impaired quality of life. Additionally, oral lesions often heal with a reduction in depth or size. This is in contrast to cutaneous lesions, which usually improve with a decrease in lesion count and show early response to treatment.

Validated scoring systems (like standardized laboratory values) are essential for objective and accurate assessment of clinical severity, prognostication of disease, deciding therapeutic options, and maintaining the homogeneity of outcome measures in clinical trials. Autoimmune Bullous Skin Disorder Intensity Score (ABSIS) (3) and Pemphigus Disease Area Index (PDAI) (4) are validated composite scales, evaluating both cutaneous and mucosal disease severity in PV. The development of a scoring system is as crucial as establishing its validity. Poor-scale construction brings into question the reliability and validity of the results even though the study is carefully planned. ABSIS and PDAI scales were developed based on the consensus reached through open discussion by a group of dermatologists with expertise and a special interest in pemphigus (4). The oral ABSIS subscale is a modified version of the previously described grading system by Saraswat and Kumar which consists of two subscales categorizing extent and severity of oral lesions (3, 5).

The Autoimmune Bullous Disease Quality Of Life (ABQOL) questionnaire was developed using standard and validated methods to measure the disease burden associated with autoimmune bullous diseases like pemphigus, bullous pemphigoid, mucous membrane pemphigoid, epidermolysis bullosa acquisita, and linear IgA bullous dermatoses (6). Low correlations were observed between ABQOL, PDAI, and ABSIS, advocating that impairment in quality of life is independent of visible disease severity (6). However, evaluation bereft of clinical disease severity assessment may not be adequate to grade a physical disease.

Oral Disease Severity Score (ODSS), which was published after the initiation of our study, has been validated for use in pemphigus patients with oral lesions (7). ODSS has its drawbacks of not being adapted from its original development for use in oral lichen planus and confusion arising out of usage of terms “erosion” and “ulceration” since the lesions cannot be deeper than the thickness of epithelium in pemphigus (8). There are no validated scoring systems that measure the severity of oral lesions exclusively except for ODSS which has drawbacks as mentioned earlier. Our study aimed to develop and validate an independent scoring system for assessment of the severity of oral lesions in PV.

## Methodology

### Settings

We conducted this study in the immunobullous disease clinic, Department of Dermatology, Postgraduate Institute of Medical Education and Research, Chandigarh, from October 2017 to November 2018. Institute Ethics Committee approved the study (INT/IEC/2017/948).

**Figure 1:**
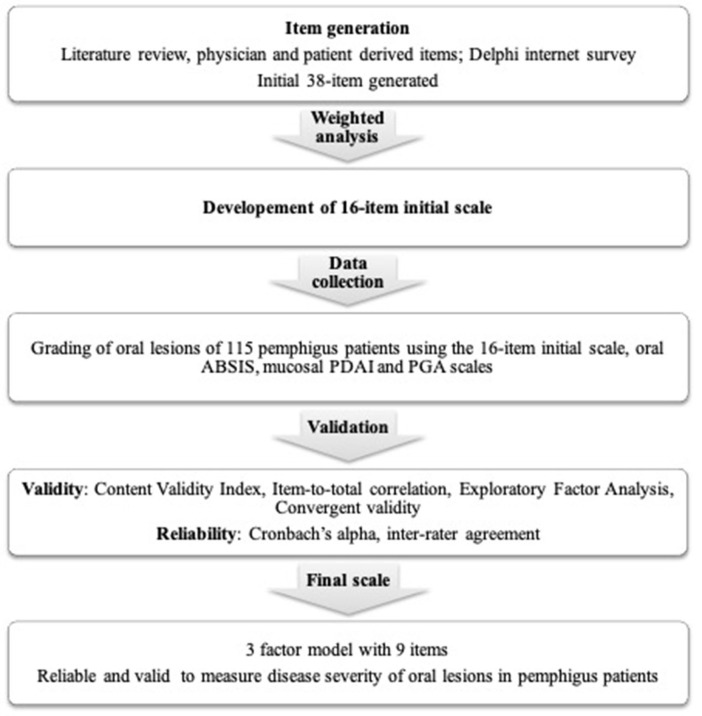
Flowchart summarizing the methods used for the development and validation of the new scoring system for oral lesions in pemphigus vulgaris.

Scale development is a multistep process (Figure 1), that includes three basic steps—item generation, data collection, and validation.

### Item Generation

We generated the initial list of candidate items for evaluating disease severity through—a methodical review of existing literature from search engines like Pubmed and Google Scholar; informal discussion with 10 practicing dermatologists; a previous study from our center on factors influencing treatment responsiveness/refractoriness of oral lesions (2); and informal discussion with 10 patients of PV having oral mucosal lesions. The list included items representing both patients' perception of disease burden and physicians' observation.

### Item Selection and Construction of the Initial Scale

As per readability and comprehensiveness, we structured 34 items to develop the initial questionnaire using https://docs.google.com/forms/u/0/. The initial questionnaire was mailed to experts (practicing dermatologists and experts in pemphigus working in India and elsewhere) for face and content validity. We maximized the follow-up reminders for expert responses with the help of reminder email followed by telephone call, if necessary. Experts rated the 34 items on a 5-point Likert scale (strongly disagree–disagree–neutral–agree–strongly agree) according to importance in determining disease severity of oral lesions in PV. They also had the option of adding new items to the list during the first round.

The four new items suggested by experts in round 1 were resent in round 2, to be rated similarly to round 1. In round 3, a questionnaire with 38 items and consolidated responses from previous rounds was sent to the same experts to reach a consensus. Only experts who completed the previous round continued to participate in subsequent rounds. Twenty-eight, 19, and 18 experts participated in rounds 1, 2, and 3, respectively. We weighted the items from (-2) to (+2) for “strongly disagree,” “disagree,” “neutral,” “agree,” and “strongly agree” categories. Finally, we developed an initial scale consisting of 16 items with score of 24 or more (maximum of 36) on weighted analysis, and we arranged the items systematically for administration in patients of PV with oral mucosal lesions.

### Data Collection

We enrolled all diagnosed and consenting patients (either sex with age ≥ 18 years) of PV with oral mucosal lesions during the study period. PV was diagnosed based on clinical findings, Tzanck smear, histopathology, and direct immunofluorescence of oral mucosal biopsy. We photographed oral lesions of all participants and graded them using the initial scale, mucosal PDAI subscale, oral ABSIS subscale, and Physician Global Assessment (PGA) scale. The mucosal PDAI subscale evaluated the number and size of erosions at 12 anatomical sites (mucous membranes), and the severity score ranged from 0 to 120. Oral ABSIS subscale included an extent score and a severity score. The extent score was based on the absence (0) or presence (1) of lesions at 11 different anatomical sites in the oral cavity. The severity score graded the discomfort associated with certain foods. The scores ranged from 0 to 11 for extent and 0 to 45 for severity equating to a total maximum score of 56 for oral ABSIS subscale. PGA of disease severity included four categories—none, mild, moderate, and severe oral mucosal disease (0–3). We did not include ODSS as it was not published when we designed our study protocol. Patient responses to the 16 items in initial scale were graded from 0 to 4 (none–mild–moderate–severe–very severe) as mentioned below.

**Figure 2:**
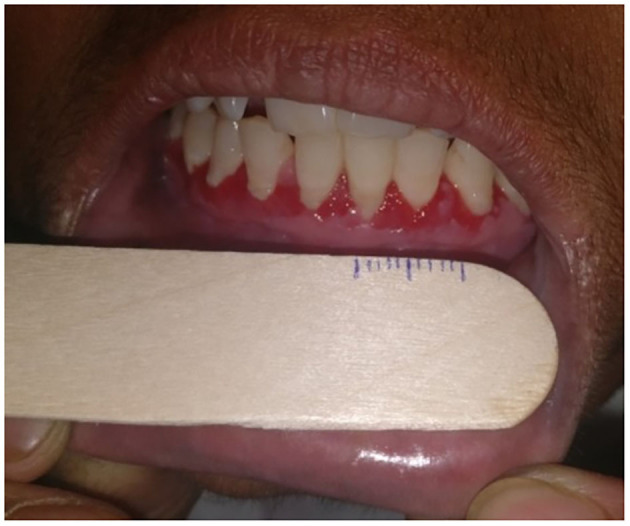
Photograph showing measurement of the size of oral erosions located on the gingiva using a ruler marked on a disposable wooden spatula.

**Figure 3:**
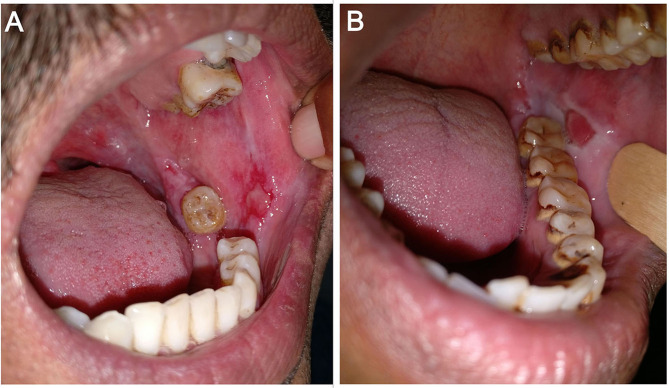
Erosions on the left buccal mucosa in two different patients—**(A)** superficial erosion and **(B)** deep erosion.

*Item(I)-1—Number of relapse(s) of the disease:* Total number of relapse(s) of the disease since onset. It can be the same as the number of relapse(s) of oral lesions in patients with disease restricted to the oral mucosa. Relapse was patient-reported and defined by the appearance of new lesions that do not heal spontaneously within 1 week in patients who were in remission, on or off therapy.
  - None: no relapse
  - Mild: single relapse
  - Moderate: 2 relapses
  - Severe: 3 relapses
  - Very severe: ≥4 relapses
*I-2—Duration of oral erosions*: the duration of the current episode of oral erosions.
  - None: ≤ 1 week
  - Mild: >1 week to 3 months
  - Moderate: >3–6 months
  - Severe: >6–12 months
  - Very severe: >12 months
*I-3—Number of relapse(s) of oral lesions*: total number of relapse(s) of oral lesions since the onset of disease irrespective of the course of cutaneous lesions. Relapse was patient-reported and defined by the appearance of new lesions that do not heal spontaneously within 1 week in patients who were in remission, on or off therapy.
  - None: no relapses
  - Mild: single relapse
  - Moderate: 2 relapses
  - Severe: 3 relapses
  - Very severe: ≥4 relapses
*I-4—Persistence of oral lesions after the subsidence of cutaneous lesions*: the duration for which oral lesions persist after the cutaneous lesions completely heal, and further new cutaneous lesions do not develop.
  - None: no persistence
  - Mild: 1–12 weeks
  - Moderate: >12–24 weeks
  - Severe: >24–48 weeks
  - Very severe: >48 weeks
*I-5—Change in size of the existing oral lesions in the last 1 week*: change, i.e., either decrease or increase in the size of oral lesions in the preceding 1 week as recalled by the patient was noted as a percentage change.
  - None: decrease by >40%
  - Mild: decrease by 30–39%
  - Moderate: decrease by 20–29%
  - Severe: decrease by 10–19%
  - Very severe: no change (or) decrease by up to 9% (or) any increase in size
*I-6—Development of new oral lesions in the last 1 week*: total number of new lesions in the preceding 1 week as recalled by the patient.
  - None: no new lesions
  - Mild: 1–3 new lesions
  - Moderate: 4–6 new lesions
  - Severe: 7–9 new lesions
  - Very severe: ≥10 new lesions
*I-7—Difficulty in eating normal food*: normal food was defined according to the individual, religious, economic, and environmental factors influencing patients' eating habits. We asked patients to rate their difficulty in eating normal food on a 5-point Likert scale—none/mild/moderate/severe/very severe.
*I-8—Difficulty in eating food according to its consistency*: We questioned patients if they had “no difficulty” (none) or difficulty in eating-raw solid diet/cooked solid diet/semisolid diet/liquid diet (mild/moderate/severe/very severe). Raw solid diet includes raw carrot, pear, peanut, and almond. Cooked solid diet includes “chapatis,” cooked mutton chicken, cooked vegetables, and steamed gram/corn. Semisolid diet consisted of porridge, “kheer,” mashed potatoes, and boiled pulses/sago. Liquid diet includes milk, buttermilk, fruit juices, and water.
*I-9—Difficulty in speaking*: we asked patients to rate their difficulty in speaking on a 5-point Likert scale—none/mild/moderate/severe/very severe.
*I-10—Difficulty in brushing teeth*: we asked patients to rate their difficulty while brushing teeth on a 5-point Likert scale—none/mild/moderate/severe/very severe.
*I-11—Difficulty in swallowing*: we asked patients to rate their difficulty in swallowing on a 5-point Likert scale—none/mild/moderate/severe/very severe.
*I-12—Restricted mouth opening*: the difficulty or discomfort associated with opening mouth fully was rated on a 5-point Likert scale—none/mild/moderate/severe/very severe.
*I-13—Number of mucosae involved*: we counted the total number of mucosal surfaces involved, viz., ocular mucosa, nasal mucosa, oral mucosa including oropharynx, genital mucosa, and anal mucosa.
  - None: no mucosal lesions
  - Mild: single mucosal involvement restricted to the oral cavity
  - Moderate: 2 mucosae involved
  - Severe: 3 mucosae involved
  - Very severe: ≥ 4 mucosae involved
*I-14—Number of sites involved in the oral cavity*: we counted the total number of sites involved in the oral cavity, viz., hard palate, soft palate, oropharynx, tongue, floor of the mouth, upper and lower gingiva, upper and lower labial mucosa, and both buccal mucosa separately (total 11 sites).
  - None: no oral lesions
  - Mild: 1 to 2 sites
  - Moderate: 3 to 5 sites
  - Severe: 6 to 8 sites
  - Very severe: 9 to 11 sites
*I-15—Overall size of erosions*: we measured the size of oral erosions (longest dimension) using a ruler manually marked on disposable wooden spatula (Figure 2), and the total sum of sizes of individual erosions was taken as the overall size of erosions/ulcers. We estimated the size of erosion(s) located in hard to reach areas (like the oropharynx) by clinical examination alone.
  - None: no oral lesions
  - Mild: up to 10 cm
  - Moderate: >10–20 cm
  - Severe: >20–30 cm
  - Very severe: >30 cm
*I-16—Depth of the erosion(s)*: As there is no standard way to define depth of erosion in the oral cavity, we gauged the depth of erosions as per the investigator's clinical judgment as either superficial or deep. It was done with the understanding of pemphigus pathogenesis that the deepest part of erosion cannot go below the basal layer of the epithelium. For example, while evaluating erosions on the buccal mucosa at almost the same location in two different patients (Figure 3), we would grade the erosion in Figure 3A as superficial and the one in Figure 3B as deep. We further counted the number of superficial and deep erosions.
  - None: no oral lesions
  - Mild: 1–10 superficial erosions
  - Moderate: 11–20 superficial erosions
  - Severe: 21–30 superficial erosions
  - Very severe: >30 superficial erosions or any number of deep erosions

### Validation

We ascertained the validity and reliability of the initial scale and eliminated items that did not meet the preset criteria if needed, to construct the final scale.

Content validity refers to the extent to which the items/scale measures the behavior for which it was intended. We assessed the content validity index (CVI) for each item by calculating the average of experts who rated the item as “agree” or “strongly agree.” CVI for the scale (S-CVI) is the average of CVI of all items. Researchers recommend an S-CVI of 0.80 or higher (9).

We assessed data quality by checking the corrected item-to-total correlation. It is the correlation between individual item and the total score. For a reliable scale, all the items should correlate with the total score. We eliminated items with correlation of <0.30 with the total scale score. “Cronbach's alpha if item is deleted” is the value to which Cronbach's alpha would increase or decrease to, following that item's deletion from the scale.

We used exploratory factor analysis (EFA) and oblimin rotation, depending on the interpretation of factor loading to evaluate the construct validity. EFA is a statistical technique within factor analysis applied to uncover the underlying structure of a relatively large set of variables or items and to identify the underlying relationship between these variables. The underlying or latent variable explaining the correlation among multiple items is known as “factor.” Bartlett's test and Kaiser–Meyer–Olkin (KMO) criterion assessed the strength of relationship among items and sampling adequacy, respectively. We used parallel analysis, scree test, and Very Simple Structure (VSS) for factor selection, as each one has its own merits and demerits.

In the factor matrix, factor loading is the correlation between items and factors, and factor loadings of 0.32 or higher meet the minimal level for interpretation of structure (10). Item's communality (*h*^2^) is the estimate of its common variance with other items, and specific variance (*u*^2^) is the unique variance not shared with other items. We deleted items having cross-loading (more than one significant factor loading) and items having low communalities (<0.40) (10). Items with low factor loadings have high unique variance that is not shared with other items and vice versa. After deleting such items, we carried out the re-specification of factor analysis to attain the final scale. We calculated absolute fit indices like root mean square error of approximation (RMSEA) and root mean square residual (RMSR) to verify how well the model fits or reproduces the data. RMSEA and RMSR values range from 0 to 1, with smaller values closer to 0.05 indicating better model fit. Tucker Lewis Index (TLI) is a relative fit index with values over 0.90 considered acceptable (11).

Convergent validity measures the correlation between two tests that are supposed to be measuring the same construct. It was measured by assessing the Spearman's rho correlation coefficients between the final scale scores, mucosal PDAI, oral ABSIS, and PGA scores. We evaluated the reliability of the final scale in terms of internal consistency using Cronbach's alpha. Cronbach's alpha values range from 0 to 1, and values between 0.60 and 0.70 are considered as the lower limit of acceptability (12). The final scale was administered in a new set of patients (*n* = 13) by two independent dermatologists (raters A and B), separately. We plotted the total scores of 13 patients using the Bland–Altman plot to assess the inter-rater agreement. We used the Psych package in R (R version 3.4.4) to analyze the data.

## Results

Response rates in the first, second, and final rounds of the Delphi questionnaire were 67.9, 94.7, and 100%, respectively. The strength of agreement between expert responses in rounds I (round 1+ round 2) and II (round 3) was measured using linear weighted kappa (*K*). The observed kappa as a proportion of maximum possible linear weighted kappa was 0.5227, indicating a moderate degree of agreement between experts (13). Kappa values of zero indicate agreement not better than chance, while positive values indicate agreement better than chance, and a negative value indicates agreement worse than chance. Despite moderate kappa for the overall agreement, we wanted to assess whether responses in desired categories such as “agree–agree” between rounds are better than responses expected by chance. The observed agreement between the experts cannot be attributed to chance alone except for the “strongly disagree–strongly disagree” category where values (expert responses) expected by chance (0.8) was marginally higher than the observed value (0) (Table 1). S-CVI of the initial scale with 16 items was 0.8 signifying strong inter-rater agreement between the experts (9).

**Table 1:** Distribution of expert responses between round I and round II.

**Expert responses**		**ROUND II**					
		**Strongly disagree**	**Disagree**	**Neutral**	**Agree**	**Strongly agree**	**Grand total**
R O U N D I	Strongly disagree	**0**	2	0	1	4	7
	Disagree	0	**11**	15	13	2	41
	Neutral	0	10	**45**	43	9	107
	Agree	1	2	25	**131**	59	218
	Strongly agree	6	0	3	55	**171**	235
Grand total		7	25	88	243	245	608
Agreement expected by chance in homogenous categories		**0.08**	**1.69**	**15.49**	**87.13**	**94.7**	199.09

*Bold values in the bottom row represent ‘expected by chance' values and bold values arranged diagonally represent the ‘observed’ values*.

### Clinical and Demographic Characteristics

**Figure 4:**
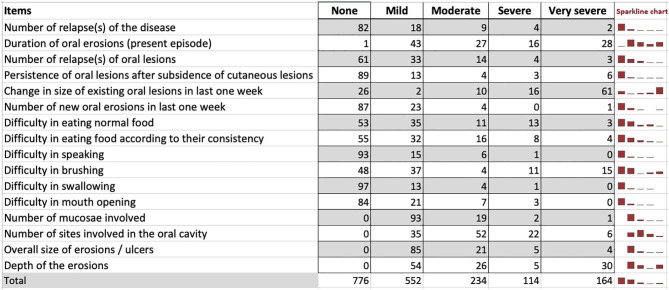
Frequencies of patient responses for each item on the initial scale.

The study cohort included 115 patients with new onset of oral erosions and those with persistent and/or recurrent oral erosions irrespective of the presence or absence of cutaneous lesions. The mean (± standard deviation) age (in years) at presentation was 42.71 ± 12.99. Women represent 62.5% of the study group. The median duration from the appearance of oral erosions to enrollment in the study was 5.5 months (IQR 2–12). We used the Sparkline chart, which is a hybrid chart having both the properties of a table (individual and accurate data) and a graph (visualize trend) to present the frequency of patient responses for each item (Figure 4). Responses to most of the items were in the “mild” to “moderate” categories except for the item “change in the size of existing oral lesions in the last 1 week,” for which most of the responses were in the “very severe” category.

**Figure 5:**
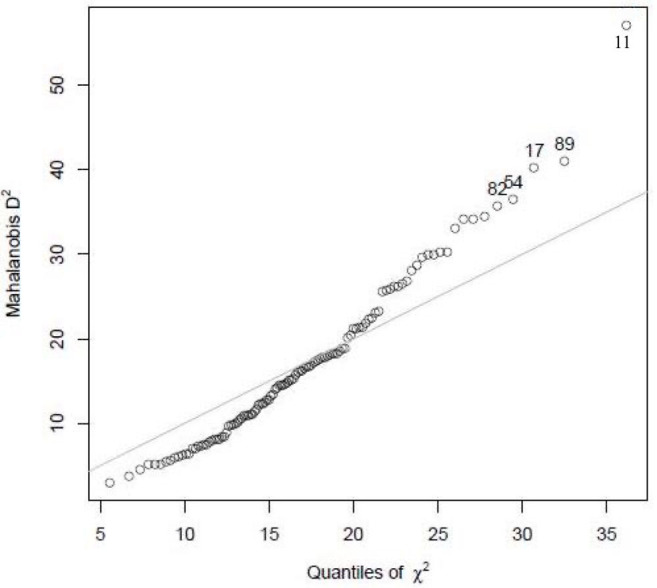
Mahalanobis graph showing outliers.

There were no missing responses for any of the items. Study participants with extreme values, i.e., outliers, were identified using the Mahalanobis distance (*D*^2^), based on a chi-square distribution. It measures how many standard deviations away a point is from the distribution in multivariate space, i.e., the relative distance between two variables with respect to the mean of the sample. Hence, the farther the variable is from the mean, the larger the *D*^2^ is. The study participant 11 lies away from the mean with higher *D*^2^ and is an outlier (Figure 5).

**Figure 6:**
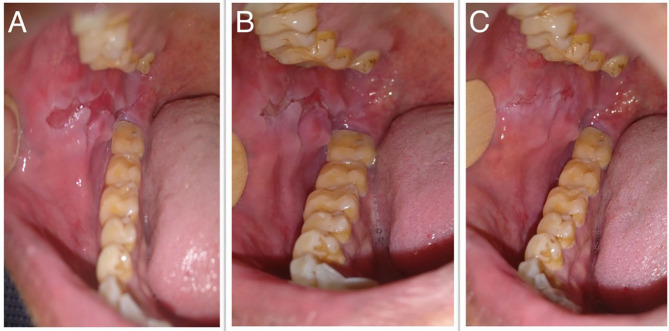
Oral erosion over the right buccal mucosa at **(A)** baseline, **(B)** 1 month, and **(C)** 2 months after starting treatment, respectively.

The median scores of oral ABSIS subscale, mucosal PDAI subscale, and PGA were 6 (IQR 3–22), 8 (IQR 4–13), and 1 (IQR 1–2), respectively. As per our clinical experience, ABSIS and mucosal PDAI scores fail to change with treatment response. In a patient with a relatively deep erosion over the buccal mucosa (Figure 6A), the ABSIS and PDAI scores were 1/11 and 1/120, respectively. With treatment, the pain or difficulty associated with eating subsided entirely within a month. ABSIS and PDAI did not capture further improvement in terms of decrease in size and depth of the erosion on monthly follow-up visits (Figures 6B,C) as the scores remained the same.

### Validation

Parallel analysis, scree plot, and VSS suggested 4, 3–5, and 4 factors, respectively. Hence, we extracted matrices with 3, 4, and 5 factors to identify the best structure. The overall measure of sampling adequacy using the KMO test was 0.74, indicating appropriateness. The Bartlett test of sphericity showed significant relationship among items (χ^2^ = 701.49; *p* < 0.001). We deleted items I-4, I-5, I-6, and I-13 as they had low loadings (<0.32) and communalities (<0.40) (also I-6 was cross loading) in the matrices and had low corrected item-total correlations (<0.30). We recalculated the corrected item-total correlations for the remaining 12 items and deleted items with correlations of <0.30- I-1, I-2, and I-3. We further performed the re-specification of factor loading for the remaining nine items.

### Factor Analysis Re-specification Results

The overall measure of sampling adequacy increased to 0.81, and the strength of the relationship among items was significant (χ^2^ = 483.59; *p* < 0.001). The appropriate number of factors to be retained as indicated by parallel analysis, scree plot, and VSS was 4, 2–4, and 3 factors, respectively. Based on factor loadings, communalities, RMSR, RMSEA, and TLI, the three-factor structure was considered a good model fit (Table 2). Items I-10, I-12, and I-16 had low communalities, yet these items were retained in the final scale as they had significant factor loadings and corrected item-total correlation.

**Table 2:** Factor analysis matrix with three factors.

**Items**	**Oral mucosal symptoms**	**Examination**	**Adjacent mucosal symptoms**	***h*^**2**^**	***u*^**2**^**
Difficulty in eating normal food (I-7)	**1.02**	0.02	−0.05	0.99	0.011
Difficulty in eating food according to their consistency (I-8)	**0.65**	0.06	0.23	0.70	0.304
Difficulty in speaking (I-9)	−0.03	0.14	**0.90**	0.88	0.117
Difficulty in brushing teeth (I-10)	**0.53**	0.09	0.06	0.37	0.630
Difficulty in swallowing (I-11)	0.21	−0.24	**0.67**	0.55	0.449
Restricted mouth opening (I-12)	**0.37**	0.11	0.26	0.38	0.618
Number of mucosae involved (I-13)^*^	–	–	–	–	–
Number of sites involved in the oral cavity (I-14)	0.10	**0.80**	0.01	0.74	0.264
Overall size of oral erosions/ulcers (I-15)	−0.04	**0.78**	0.06	0.62	0.379
Depth of the oral erosion(s) (I-16)	0.12	**0.49**	−0.05	0.28	0.720
RMSR = 0.03; RMSEA index = 0.086; TLI = 0.939					

“*I” represents item number on the initial scale*.

* *Items which were deleted after weighted analysis*.

*h^2^, communality/common variance; u^2^, unique variance; RMSEA, root mean square error of approximation; RMSR, root mean square residual; TLI, Tucker Lewis Index*.

*Bold values represent significant factor loadings i.e., factory loading > 0.32*.

Based on the factor loadings (Table 2), we grouped the final nine items under three distinct variables—symptoms due to oral mucosal involvement (4 items), symptoms due to adjacent mucosal involvement (2 items), and features observed on clinical examination (3 items). Each item in the final scale—Pemphigus Oral Lesions Intensity Score (POLIS)—was graded from 0 to 4 using simple weighting technique (14) (Table 3), adding up to a total scale score of 36.

**Table 3:** Pemphigus Oral Lesions Intensity Score (POLIS) with 9 items.

**Items**	**Points**				
	**0**	**1**	**2**	**3**	**4**
**Symptoms related to oral cavity**					
Difficulty in eating normal food^a^	None	Mild	Moderate	Severe	Very severe
Difficulty in eating food according to consistency^b^	None	Raw solids	Cooked solids	Semisolids	Liquids
Difficulty in brushing	None	Mild	Moderate	Severe	Very severe
Difficulty in mouth opening	None	Mild	Moderate	Severe	Very severe
**Symptoms related to other mucosae**					
Difficulty in swallowing	None	Mild	Moderate	Severe	Very severe
Difficulty in speaking	None	Mild	Moderate	Severe	Very severe
**Oral cavity examination**					
Number of sites involved in the oral cavity (maximum 11)^c^	0	1–2	3–5	6–8	9–11
Overall size of the erosions/ulcers^d^	0	Up to 10 cm	>10–20 cm	>20–30 cm	>30 cm
Depth of the erosions^e^	0	1–10 superficial erosions	11–20 superficial erosions	21–30 superficial erosions	>30 superficial erosions/any number of deep erosions

*Minimum score = 0; maximum score = 36*.

a *Normal food is defined according to the individual, religious, economic, and environmental factors influencing patients' eating habits*.

b *Raw solid diet includes raw carrot, pear, peanut, and almond. Cooked solid diet includes “chapatis,” cooked mutton/chicken, cooked vegetables, and steamed gram/corn. Semisolid diet consisted of porridge, “kheer,” mashed potatoes, and boiled pulses/sago. Liquid diet includes milk, buttermilk, fruit juices, water, etc*.

c *Oral cavity is divided into hard palate, soft palate, oropharynx, tongue, floor of mouth, upper and lower gingiva, upper and lower labial mucosa, and both buccal mucosa separately*.

d *Total sum of sizes of individual erosions was taken as the overall size of erosions/ulcers*.

e *The total number of superficial and deep erosions were counted*.

*Copyright © 2020 Sindhuja, De, Handa, Goel, Mahajan and Kishore*.

The minimum and maximum possible values for POLIS, oral ABSIS subscale, mucosal PDAI subscale, and PGA scales were 0–36, 0–56, 0–120, and 0–3, respectively. The corrected scores for each scale were used in the analysis [corrected score = (maximum score-observed score)/maximum score] to maintain uniformity. POLIS scale with 9 items showed very strong correlation with oral ABSIS subscale (*r* = 0.85, *p* < 0.001), strong correlations with mucosal PDAI (*r* = 0.70, *p* < 0.001), and PGA (*r* = 0.60, *p* < 0.001).

### Reliability

**Figure 7:**
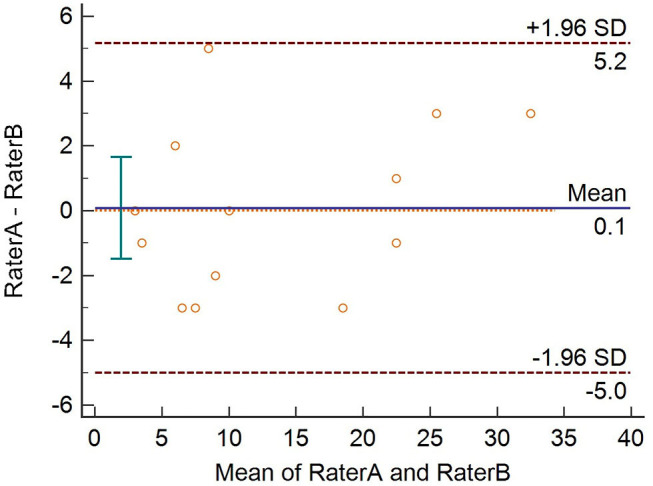
Bland–Altman plot showing strong inter-rater agreement.

Cronbach's alpha of POLIS was 0.857, suggesting that the items have good internal consistency. We used the item-deleted alpha analysis to assess whether or not the exclusion of any particular item would increase the internal consistency reliability of the scale (Table 4). All items in POLIS had correlations higher than 0.30 (Table 4). We used the Bland–Altman plot to estimate the inter-rater agreement as the inter-rater kappa agreement cannot be applied to continuous data. It is a scatter plot of the difference between the two measurements by raters A and B (Y-axis) against the average of the two measurements (X-axis). It provides a graphical display of the mean difference between the two raters or techniques with 95% agreement limits. All points in the plot were positioned within ± 2 SD, indicating a strong agreement between the raters (Figure 7).

**Table 4:** Results of item-deleted alpha analysis.

**Items**	**Corrected item–total correlation**	**Cronbach's alpha if item deleted**
Difficulty in eating normal food	0.765	0.791
Difficulty in eating food according to consistency	0.719	0.797
Difficulty in speaking	0.660	0.816
Difficulty in brushing	0.546	0.828
Difficulty in swallowing	0.456	0.831
Difficulty in mouth opening	0.562	0.820
Number of sites involved in the oral cavity	0.606	0.814
Total size of the oral erosions	0.498	0.825
Depth of the oral erosions	0.390	0.844

## Discussion

Owing to the lack of validated, independent scoring system for oral lesions that can precisely reflect disease activity, a 9-item scale (Pemphigus Oral Lesions Intensity Score, POLIS) was developed and validated for oral lesions in pemphigus. A three-factor structure emerged, capturing features of both patients' perspective and clinicians' assessment of disease severity. The study analyses provided adequate evidence of internal consistency, content, construct and data validity.

Though validated, ABQOL does not assess clinical disease severity and is not specific for oral lesions of pemphigus. Using two scoring systems, i.e., ABQOL for assessing quality of life and PDAI/ABSIS for clinical disease activity increases the time required for evaluation. The strength of the new scale is that it includes 6 items which directly addresses quality of life impairment due to oral erosions and 3 items for assessing clinical disease severity.

A significant strength of this study is the use of qualitative methods to generate items for evaluating oral lesions in pemphigus. The Delphi method was used for initial item generation as it minimizes the biasing effects of dominant individuals, irrelevant discussions, and group pressure toward agreement as is seen in group discussions (15). The Internet-based Delphi questionnaire was chosen to reduce the time, costs, problems in communication, and participant attrition. A structured questionnaire was used in all the rounds to save time and decrease the dropout rate. The study cohort included both new and follow-up patients incorporating a relatively large set of pemphigus patients with most of them having mild to moderate disease burden.

In POLIS, patient-perceived symptoms like difficulty in eating, brushing teeth, speaking, swallowing, and mouth opening were found to be important in grading severity of oral lesions. While the symptoms associated with eating had been addressed in oral ABSIS subscale, symptoms associated with other activities mentioned above were not included. Gingival erosions are often associated with difficulty in brushing rather than difficulty in eating. Extensive or deep erosions over the buccal mucosa particularly over the retromolar trigone and labial commissures are associated with difficulty in mouth opening. Difficulty in speaking and swallowing indicates involvement of the larynx, pharynx, and esophagus (adjacent mucosae) and thereby disease extent and severity.

The mucosal component of PDAI includes the number and size of erosions at predefined anatomical locations while the oral involvement score of ABSIS includes the number of sites involved in the oral cavity and symptoms of discomfort associated with eating or drinking. We observed that these variables were not adequate to capture the clinical variability of oral erosions over time (Figure 6). POLIS includes size and depth of erosions and accounts for these changes.

Along with the number of sites involved in the oral cavity, POLIS also includes the number of erosions, further categorizing them as superficial or deep. Presence of any number of deep erosions was considered as severe disease as they are relatively refractory to treatment (2). By incorporating both size and number of erosions, POLIS effectively assesses the clinical severity of oral lesions in PV where erosions tend to coalesce and extend particularly over the gingival margins. The size and number of erosions were graded at intervals of 10 cm and 10 erosions, respectively, which makes POLIS simpler and comfortable to use. POLIS showed better correlation with oral ABSIS than with mucosal PDAI, probably owing to assessment of patient-perceived symptoms along with clinical severity in the oral ABSIS subscale.

Considering the drawbacks of the existing scales and the use of standard statistical methods in the development and validation of POLIS, it can be considered as a useful tool for the accurate assessment of severity of oral lesions in PV. Limitations of the study include study cohort from a single center, absence of data regarding usability, and time taken to administer the scale. Further studies evaluating the correlation between POLIS, ODSS, and ABQOL are required.

## Conclusion

Due to the remarkable clinical variability of oral lesions in pemphigus, several parameters are needed to precisely reflect disease activity. Given the absence of disease severity scoring systems developed using standard and objective methods at the time of initiation of our study, we report a new disease severity scale for assessing severity of oral lesions in pemphigus. It combines both clinical disease severity and quality of life assessment, thus measuring the severity of oral mucosal PV holistically. The scales' responsiveness to change is being assessed, and the results will be published subsequently in another study.

## Data Availability Statement

The raw data supporting the conclusions of this article will be made available by the authors, without undue reservation.

## Ethics Statement

The studies involving human participants were reviewed and approved by Institutional ethics committee Post Graduate Institute of Medical Education and Research #INT/IEC/2017/948. The patients/participants provided their written informed consent to participate in this study.

## Author Contributions

TS and KK: had full access to all the data in the study and took responsibility for the integrity of the data, and the accuracy of the data analysis. TS, DD, SG, and KK: study concept and design. TS, DD, SH, RM, SG, and KK: acquisition, analysis, or interpretation of data. TS: drafting of the manuscript. DD, SH, SG, KK, and RM: critical revision of the manuscript for important intellectual content, administrative, technical, or material support, and study supervision. KK and SG: statistical analysis. All authors contributed to the article and approved the submitted version.

## Conflict of Interest

The authors declare that the research was conducted in the absence of any commercial or financial relationships that could be construed as a potential conflict of interest.
